# Prevalence, determinants, and outcomes of unintended pregnancy in Sohag district, Egypt

**DOI:** 10.1186/s42506-019-0014-9

**Published:** 2019-03-13

**Authors:** Eman Abd-El Baset Mohamed, Ahmed Fathy Hamed, Fouad M. A. Yousef, Esraa Aly Ahmed

**Affiliations:** Public Health and Community Medicine Department, Sohag Faculty of Medicine, Sohag University, Sohag, Egypt

**Keywords:** Unintended pregnancy, Prevalence, Determinants and outcomes

## Abstract

**Background:**

Unintended pregnancies may be mistimed or unwanted. It represents 40% of all pregnancies, and it had multiple risky health outcomes. It is essential to understand the factors affecting unintended pregnancies and their consequences to develop strategies that help prevent them. The present study is conducted to identify the prevalence, determinants, and outcomes of unintended pregnancies in Sohag district, Egypt.

**Methods:**

A cross-sectional study was conducted in Sohag district, 2016. Five hundred fifty-four ever married women aged 18–49 years whose last pregnancy was in the 3 years preceding the data collection date were randomly selected from rural and urban localities. Data were collected through home visits using a validated questionnaire. In this study, Sohag city represents the urban place, while Tunis and El-Sheikh Makram villages represent the rural places.

**Results:**

Nearly one third (30.7%) of the study sample had an unintended pregnancy within the last 3 years from the time of interview. Regression analysis showed that young women < 30 (OR = 2.24, 95% CI 1.12–4.48, *p* = 0.02), young husbands ≤ 30 (OR = 5.44, 95% CI 1.14–26.11, *p* = 0.03), women working for cash (OR = 6.16, 95% CI 3.15–13.92, *p* < 0.0001), monthly income ≤ 1200 LE (OR = 34, 95% CI 6.41–187.52, *p* < 0.0001), and spacing < 24 months (OR = 8.79, 95% CI 4.33–17.80, *p* < 0.0001) were risk factors for mistimed pregnancy. On the other hand, women working for cash (OR = 11.43, 95% CI 3.22–40.62, *p* < 0.0001), living children ≥ 5 (OR = 11.45, 95% CI 2.84–46.07, *p* = 0.001), and the woman’s perception of her family size as higher than the ideal (OR = 394.8, 95% CI 97.36–1601.17, *p* < 0.0001) were risk factors for unwanted pregnancy. Mistimed and unwanted pregnancies were significantly associated with late start of antenatal care (ANC), low birth weight (LBW), and no breastfeeding. In addition, unwanted pregnancies were associated with more pregnancy complications.

**Conclusions and recommendations:**

Unintended pregnancy represents a public health problem in Sohag. Therefore, improving services in rural areas and improving the economic level and effective use of family planning methods could reduce the risks associated with the unintended pregnancy.

## Introduction

Pregnancy and its related problems contribute to a significant proportion of reproductive mortality with maternal mortality is unacceptably high. About 830 women die from pregnancy or its related complications around the world every day [1]. Unintended pregnancy is an important worldwide public health problem. It affects not only women, but it affects their families and society, as well. Worldwide, each year there are about 80 million women experiencing unintended pregnancy [2], which includes mistimed and unwanted pregnancy. While the former means that a woman gets pregnant before she wants, the latter is the occurrence of pregnancy when no children were desired [3]. Accordingly, every married woman is at risk of the problem of unintended pregnancy.

The unintended pregnancies prevalence was 40% in 2012 with the largest proportion occurred in Africa [4]. Over the past decade, unintended pregnancy prevalence rate ranged from 15 to 58% of pregnancies in the countries of North Africa and the Middle East. Its prevalence rate was estimated as 58% in Yemen, 38% in Palestine, 32% in Morocco, and 31% in Syria and Algeria. In Egypt, it was estimated to be 23% [5].

One of the serious results related to unintended pregnancy is abortion [1]. In addition, unintended pregnancy has multiple risky health outcomes, such as the decreased likelihood of breastfeeding initiation and continuation [6].

The Egyptian Demography Health Survey (EDHS), 2014, [7] showed that, overall, 16% of births in the 5-year period were not wanted at the time of conception (i.e., including the mistimed and unwanted). Among the births not wanted at the time of conception, just over half (8% of all births) were not wanted at all. There was a gap between the total fertility rate and the wanted fertility rate about 0.8 births and concluded that, if unwanted births could be eliminated, the total fertility rate in Egypt would decline by 20%. There is no available data about unintended pregnancy in Sohag. The present study was conducted to identify the prevalence, determinants, and outcomes of unintended pregnancies in Sohag district, Egypt.

## Subjects and methods

### Study design

This is a cross-sectional study.

### Sample size

Using a prevalence of 23% [5], a sample of 273 women is required. However, 554 women were included in this study.

### Data collection

Sohag governorate consists of 12 districts. The biggest one is Sohag district. Sohag district consists of Sohag city and 32 villages. Sohag city was selected to represent the urban area, and two rural areas near the city (Tunis and El-Sheikh Makram villages) were selected to represent the rural area. Data was collected from women through a household survey.

### Inclusion and exclusion criteria

The inclusion criteria include ever married women aged 18–49 years.

The exclusion criteria include women with last pregnancy for more than 3 years, to avoid recall bias as mother-kid relationship may be an obstacle in calling this child as unwanted and inability to obtain informed consent.

### Study tool

The researcher used a questionnaire that was validated and used by a previous related study in Helwan district [8]. It has four sections: section (1) the sociodemographic characteristics, section (2) obstetric and reproductive history, section (3) the history of last pregnancy, and section (4) outcomes of the most recent pregnancy. A pilot study was conducted to detect any problem that might face the researchers during the study.

### Data analysis

Analysis was carried using the SPSS software (SPSS for Windows, Version 16.0. Chicago, SPSS Inc.). Chi-square test was done to compare the different groups. Univariate and multivariate logistic regression was used to detect the determinants of unintended pregnancies (only the final model was shown in the results). Low-risk groups in our result were used as the reference group in the logistic regression analysis. *P* value was considered significant if below 0.05.

## Results

**Fig. 1:**
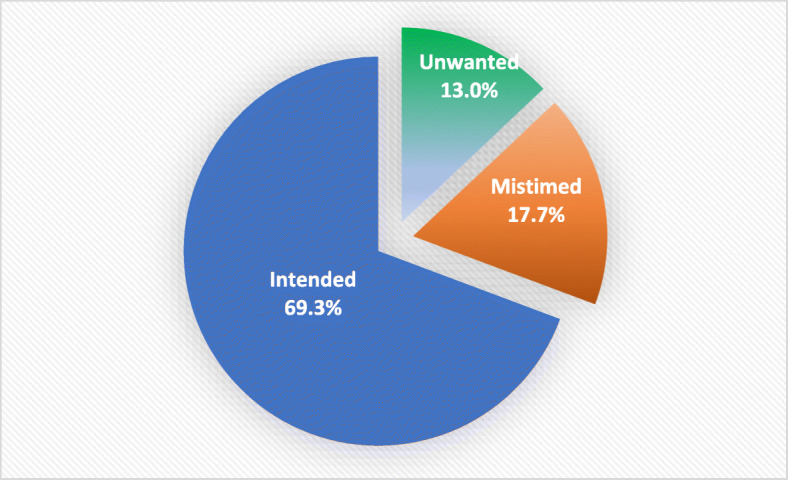
Pregnancy intention status among women in Sohag district, 2016

Five hundred and fifty-four women aged 18–49 years participated in this study, of whom 72 women (13.0%) reported that their most recent pregnancy was unwanted and 98 (17.7%) claimed that their pregnancy was mistimed. The other pregnancies 384 (69.3%) were planned (Fig. 1).

Figure 1 shows the pregnancy intention among women, and Tables 1 and 2 show the distribution of type of pregnancies (planned, unintended, and missed) according to different characteristics of studied women. The prevalence of unwanted pregnancies was significantly higher in women aged more than 40 years (54.5%), husband aged 41–50 years old (43.0%), women with five or more deliveries (56.2%), women who had two or more abortions (36.2%), those who had five or more living children (61.4%), women with more than 48 months pregnancy spacing (31.5%), women with family size more than the ideal family size (as believed by women) (93.8%), and those who had four or more boys (43.8%). The mistimed pregnancy was significantly more common in women aged less than 20 years old (27.8%), husband age 50 or more (33.3%), women working for cash (25.2%), and pregnancy spacing less than 24 months (35.3%).

**Table 1:** Pregnancy intention status in relation to women’s sociodemographic factors, Sohag district, Egypt, 2016

Parameter	Intended No. (%)	Mistimed No. (%)	Unwanted No. (%)	Total *N* = 554 No. (%)	*p* value
Women age					
≤ 20	26 (72.2)	10 (27.8)	0 (0)	36 (6.5)	< 0.0001*
21–30	224 (72.0)	72 (23.2)	15 (4.8)	311 (56.1)	
31–40	129 (65.8)	82 (8.2)	51 (26.0)	196 (35.4)	
> 40	5 (45.5)	0 (0)	6 (54.5)	11 (2.0)	
Husband age					
≤ 30	143 (83.1)	29 (16.9)	0 (0)	172 (31.1)	< 0.0001*
31–40	192 (64.7)	67 (22.5)	38 (12.8)	297 (53.5)	
41–50	45 (57.0)	0 (0)	34 (43.0)	79 (14.3)	
≥ 50	4 (66.7)	2 (33.3)	0 (0)	6 (1.1)	
Women education					
Illiterate	49 (67.1)	16 (21.9)	8 (11)	73 (13.2)	0.01*
Read and write	45 (55.6)	17 (21)	19 (23.4)	81 (14.6)	
Essential education	164 (69.5)	39 (16.5)	33 (14.0)	236 (42.6)	
Secondary or higher	126 (76.8)	26 (15.9)	12 (7.3)	164 (29.6)	
Husband education					
Illiterate	54 (68.4)	14 (17.7)	11 (13.9)	79 (14.3)	0.02*
Read and write	33 (58.9)	19 (33.9)	4 (7.1)	56 (10.1)	
Essential education	148 (70.1)	38 (18.0)	25 (11.9)	211 (38.1)	
Secondary or higher	149 (71.6)	27 (13.0)	32 (15.4)	208 (37.5)	
Women work					
Not working for cash	310 (73.4)	64 (15.1)	49 (11.6)	423 (76.4)	0.001*
Working for cash	74 (56.5)	33 (25.2)	24 (18.3)	131 (23.6)	
Husband occupation					
Does not work	5 (80.0)	0 (0)	1 (20.0)	5 (0.9)	0.30
Manual/skilled	227 (68.6)	65 (19.6)	39 (11.8)	331 (59.7)	
Employee	72 (64.5)	22 (20.0)	16 (14.5)	110 (19.9)	
Professional	81 (75.0)	11 (10.2)	16 (14.8)	108 (19.5)	
Residence					
Urban	187 (74.5)	33 (13.6)	30 (12.0)	251 (45.3)	0.03*
Rural	197 (65.0)	64 (21.1)	42 (13.9)	303 (54.7)	
Family type					
Nuclear	139 (65.5)	40 (18.9)	33 (15.6)	212 (38.3)	0.28
Extended	245 (71.6)	57 (17.0)	40 (11.4)	342 (61.7)	
Monthly income					
≤ 1200 LE	157 (69.2)	48 (21.1)	22 (9.7)	227 (41.0)	0.02*
1200–3000 LE	186 (67.2)	48 (17.3)	43 (15.5)	277 (50.0)	
> 3000 LE	41 (82.0)	2 (4.0)	7 (14.0)	50 (9.0)	

Percentage are represented as row percentage except for the total column percentage

*Significant at *p* < 0.05

**Table 2:** Pregnancy intention status in relation to some women’s reproductive variables, Sohag district, Egypt, 2016

Parameter	Intended No. (%)	Mistimed No. (%)	Unwanted No. (%)	Total No. (%)	*p* value
Deliveries					
0–2	177 (76.6)	54 (23.4)	0 (0)	231 (41.7)	< 0.0001*
3–4	177 (70.8)	42 (16.8)	31 (12.4)	250 (45.1)	
≥ 5	30 (41.1)	2 (2.7)	41 (56.2)	73 (13.2)	
Abortions					
0	278 (69.8)	79 (19.9)	41 (10.3)	398 (71.8)	< 0.0001*
1	76 (77.6)	12 (12.2)	10 (10.2)	98 (17.7)	
≥ 2	30 (51.7)	7 (12.1)	21 (36.2)	58 (10.5)	
Living children					
0–2	177 (76.6)	54 (23.4)	0 (0)	231 (41.7)	< 0.0001*
3–4	184 (72.7)	34 (15.8)	30 (11.5)	253 (45.7)	
≥ 5	23 (32.9)	4 (5.7)	43 (61.4)	70 (12.6)	
Spacing					
≤ 24 months	128 (53.8)	84 (35.3)	26 (10.9)	238 (51.1)	< 0.0001*
24–48 months	120 (77.4)	12 (7.4)	23 (14.8)	155 (33.3)	
> 48 months	50 (68.5)	0 (0)	23 (31.5)	73 (15.6)	
Achieved family size					
Ideal	125 (73.1)	33 (19.3)	13 (7.6)	171 (31.0)	< 0.0001*
Less than the ideal	255 (79.9)	64 (20.1)	0 (0)	319 (57.8)	
More than the ideal	4 (6.2)	0 (0)	60 (93.8)	62 (11.2)	
Gender of living children					
Males only	72 (83.7)	12 (14.0)	3 (2.3)	86 (15.8)	< 0.0001*
Females only	87 (81.3)	20 (18.7)	0 (0)	107 (19.7)	
Both	217 (61.8)	64 (18.2)	70 (19.9)	351 (64.5)	
Boys					
0–1	226 (76.6)	61 (20.7)	8 (2.7)	295 (54.2)	< 0.0001*
2–3	141 (60.5)	35 (15.0)	57 (24.5)	233 (42.8)	
≥ 4	9 (56.2)	0 (0)	7 (43.8)	16 (2.9)	
Woman age at first pregnancy					
≤ 20	167 (71.1)	35 (14.9)	33 (14.0)	235 (42.4)	0.50
21–25	141 (66.2)	44 (20.7)	28 (13.1)	213 (38.4)	
26–30	73 (72.3)	17 (16.8)	11 (10.9)	101 (18.2)	
> 30	3 (60)	2 (40.0)	0 (0)	5 (0.9)	
Knowledge about menstrual cycle					
Yes	104 (74.8)	26 (18.7)	9 (6.5)	139 (25.1)	0.03*
No	280 (67.5)	72 (17.3)	63 (15.2)	415 (74.9)	

Percentages are represented as row percentage except for the total column percentage

*Significant at *p* < 0.05

The results of the final model of factors determining the occurrence of missed and unwanted pregnancy were shown in Table 3. Risk factors for missed pregnancy were young women age < 30 (OR = 2.24, 95% CI 1.12–4.48, *p* = 0.02), young women age < 30 (OR = 5.44, 95% CI 1.14–26.11, *p* = 0.03), women working for cash (OR = 6.16, 95% CI 3.15–13.92, *p* < 0.0001), monthly income ≤ 1200 LE (OR = 18, 95% CI 3.70–94.33, *p* < 0.0001) or monthly income (1200–3000 LE) (OR = 34, 95% CI 6.41–187.52, *p* < 0.0001), and spacing < 24 months (OR = 8.79, 95% CI 4.33–17.80, *p* < 0.0001). Risk factors for unwanted pregnancy were women working for cash (OR = 11.43, 95% CI 3.22–40.62, *p* < 0.0001), living children ≥ 5 (OR = 11.45, 95% CI 2.84–46.07, *p* = 0.001), and reporting more than the ideal family (OR = 394.8, 95% CI 97.36–1601.17, *p* < 0.0001).

**Table 3:** Results of the final model of logistic regression analysis of determinants of missed and unwanted pregnancy in women of reproductive age, Sohag, Egypt, 2016

Parameter	Adjusted odds ratio (95% CI)	*p* value
Determinants of mistimed pregnancy		
Women age#		
> 30	1	
≤ 30	2.24 (1.12–4.48)	0.02*
Husband age#		
> 30	1	
≤ 30	5.44 (1.14–26.11)	0.03*
Women work		
Not working for cash	1	
Working for cash	6.16 (3.15–13.92)	< 0.0001*
Monthly income		
> 3000 LE	1	
1200–3000 LE	18 (3.70–94.33)	
≤ 1200 LE	34 (6.41–187.52)	
Spacing #		
> 24 m	1	
≤ 24 m	8.79 (4.33–17.80)	< 0.0001*
Determinants of unwanted pregnancy		
Women work		
Not working for cash	1	
Working for cash	11.43 (3.22–40.62)	< 0.0001*
Living children#		
≤ 4	1	
≥ 5	11.45 (2.84–46.07)	0.001*
Achieved family size#		
Less than ideal or ideal	1	
More than the ideal	394.8 (97.36–1601.17)	< 0.0001*

*Significant at *p* < 0.05

#Some categories are combined to avoid zero observation in some categories

**Fig. 2:**
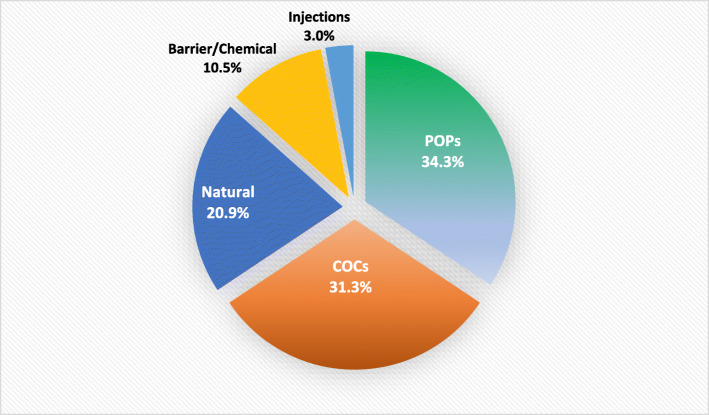
Contraceptive failure among women with unintended pregnancy in Sohag district, 2016

Of those women who had an unintended pregnancy, 61 (35.9%) were using contraceptive methods when they conceived. Thus, pregnancy happened due to method failure. Most of the failure rate is due to pill usage either combined or progesterone only (31.3% and 34.3%), followed by natural methods (20.9%) and chemical and barrier methods (10.5%). The least ranked were injectable methods (2.99%) (Fig. 2).

**Fig. 3:**
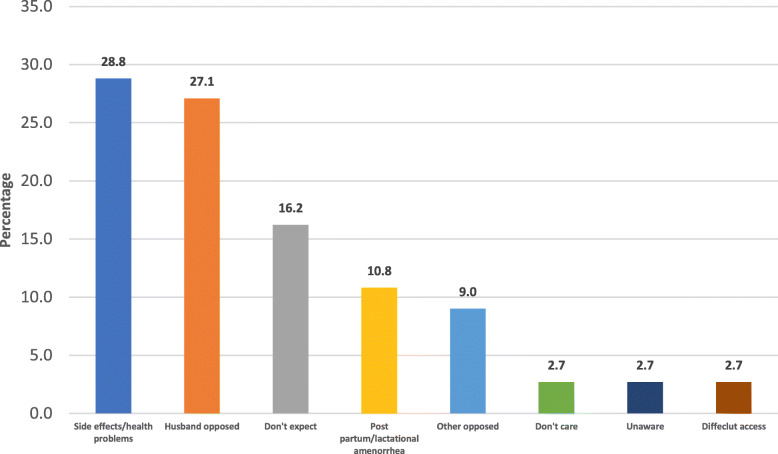
Causes of non-use of contraceptive methods among women with unintended pregnancy, Sohag district, 2016

Sixty-four percent of women who had unintended pregnancy did not use contraceptive methods. The most common cause of non-using any contraceptives among women having unintended pregnancy was side effects or health problems (28.8%). The second common cause is husband refusal (27.0%) followed by no expectancy that pregnancy can happen (16.2%) (Fig. 3).

Table 4 shows the outcome of pregnancies. Compared to intended pregnancy, the mistimed and unwanted pregnancy was significantly associated with late start of antenatal care (ANC) (39.8% and 43.1% respectively), low birth weight (LBW) (22.0% and 15.7% respectively), and no breastfeeding at all (24.2% and 12.9 respectively). Unwanted pregnancy was also associated with occurrence of complications of pregnancy (34.7%).

**Table 4:** Relation between pregnancy intention and pregnancy outcomes, Sohag district, Egypt, 2016

Parameter	Intended No. (%)	Mistimed No. (%)	Unwanted No. (%)	Total (*n* = 554) No. (%)	*p* value
ANC					
< 4	33 (8.6)	13 (13.3)	3 (4.2)	49 (8.8)	0.11
≥ 4	351 (91.4)	85 (86.7)	69 (95.8)	505 (91.2)	
Start of ANC					
In the first trimester	329 (85.7)	59 (60.2)	41 (56.9)	429 (77.4)	< 0.0001*
After that	55 (14.3)	39 (39.8)	31 (43.1)	125 (22.6)	
Complications					
No	325 (84.6)	80 (81.6)	47 (65.3)	452 (81.6)	0.001*
Yes	59 (15.4)	18 (18.4)	25 (34.7)	102 (18.4)	
Place of delivery					
Hospital	348 (90.6)	91 (92.9)	57 (79.2)	496 (89.5)	0.007*
Home	36 (9.4)	7 (7.1)	15 (20.8)	58 (10.5)	
Pregnancy outcome					
Abortion	15 (3.9)	7 (7.1)	2 (2.8)	24 (4.3)	0.14
Live birth	359 (93.5)	91 (92.9)	70 (97.2)	520 (93.9)	
Dead birth	10 (2.6)	0 (0)	0 (0)	10 (1.8)	
Duration (*n* = 520)#					
Term	324 (90.3)	85 (93.4)	61 (87.1)	470 (90.4)	0.41
Preterm	35 (9.8)	6 (6.6)	9 (16.9)	50 (9.9)	
Birth weight (*n* = 520) #					
LBW	38 (10.6)	20 (22.0)	11 (15.7)	69 (13.3)	0.01*
Average	321 (89.4)	71 (78.0)	59 (84.3)	451 (86.7)	
Incubator admission (*n* = 520) #					
Yes	89 (24.8)	32 (35.2)	16 (22.9)	137 (26.3)	0.14
No	270 (75.2)	59 (64.8)	54 (77.1)	383 (73.7)	
Breastfeeding					
Yes	328 (91.4)	69 (75.8)	61 (87.1)	458 (88.1)	< 0.0001*
No	31 (8.6)	22 (24.2)	9 (12.9)	62 (11.9)	
Initiation of breastfeeding					
Within the first hour	220 (67.1)	52 (75.4)	44 (72.1)	316 (69.0)	0.34
After that	108 (32.9)	17 (24.6)	17 (27.9)	142 (31.0)	

Percentages are represented as column percentage

*Significant at *p* < 0.05

#The total does not sum up to 554 due to occurrence of 24 abortions and 10 deaths

## Discussion

The current study revealed that the prevalence of unintended pregnancy in Sohag district is 30.7%. This is higher than the rates reported by previous studies in Egypt. The EDHS 2014 [7] showed a prevalence of 16% for mistimed and unwanted together. A study in Beheira Governorate, Egypt, (2002) reported a rate of 23.6% [9]. The difference could be attributed to the different socioeconomic factors and use of contraceptive methods. The current rate in this study may be even higher due to the tendency of Egyptian women after the children are born to avoid declaring that they were unwanted.

Prevalence of unintended pregnancy differs from one community to another. In Egypt, it is much less than that of the USA which is about 45% [10] in which the prevalence of adolescent pregnancy is high and pregnancy in unmarried women which is rare in our community. Also, the prevalence is close to other Islamic countries, e.g., the prevalence of unintended pregnancy in Iran is about one third [11]. However, the unintended pregnancy rate in some African countries is lower, e.g., Nigeria rated 18.2% [12].

Women in rural areas were more liable to unintended pregnancy than those in urban areas. This result disagreed with another study conducted in Sudan [13] which reported no significant difference between urban and rural women. Our finding may be due to difference in educational level as only 15% of women in the rural area were educated up to the secondary or higher level compared to 47% in the urban area. Education affects the awareness of women regarding using contraceptive methods effectively and seeking medical care when any complication happens. In Sohag district, educated women—secondary school or higher—experience unwanted pregnancy less than illiterate women or those who just read and write. Studies in Ethiopia [14, 15] also reported a similar finding. Unintended pregnancy did not differ significantly by different husband occupation categories in our study. Shaheen et al. in Egypt found that only agricultural work increased the risk of unintended pregnancy significantly [16]. This may be due to a lower level of education among the rural and agricultural area.

The birth interval of the recent birth was significantly associated with pregnancy intentions. Women who spaced the pregnancy of their most recent birth by less than 1 year were more likely than those who did so by more than 1 year to report that their pregnancy had been mistimed reaching zero percentage in those who spaced more than 2 years. In contrast, women who spaced by more than 2 years in Tehran were more likely to report their pregnancy as unwanted than women who spaced by less than 2 years [17].

Among women who did not use contraceptives, side effects or fear of side effects was the most common cause for non-use of a method reflecting the high need of our district to health education. The next cause for non-use was husband refusal, reflecting a decrease of women autonomy in our society. The same applies to African counties as reported by Sedgh et al. [18].

A further in-depth statistical analysis using multivariate logistic regression revealed that young women with age < 30, young husband with age < 30, women working for cash, low monthly income, and close spacing were risk factors for mistimed pregnancy. On the other hand, women working for cash, living children ≥ 5, and the woman’s perception of her family size as higher than the ideal were risk factors for unwanted pregnancy. In Canada, it was found that young age, low income, and high school education are the final risk factors for unintended pregnancy [19].

This study also highlights some prenatal and perinatal outcomes, including antenatal care. There was no significant difference between pregnancy intention status and the number of ANC visits. This disagrees with results of a study conducted in southwestern Ethiopia and reported that women with unintended pregnancies were less likely to receive adequate antenatal care as compared to those with intended pregnancies [20]. The authors found an increased risk of complications among women with an unintended pregnancy. These findings were also found from another study in Upper Egypt [21]. A study, however, did not reveal a relationship between pregnancy intention status and maternal complications during pregnancy [22]. In the present population, there was no difference in the time of delivery (term or preterm) with different pregnancy intention categories. This study showed high prevalence of LBW among miss timed and unwanted pregnancies. Hidden factors like decreased vitamin intake, having frequent births, or increased age of the mother may be the true causes of LBW rather than pregnancy intention, as shown in other studies [23, 24].

### Study limitations

This study discusses only the problem of unintended pregnancy from the women’s view. It is also limited to the pregnancy within the last 3 years from interview. There is a possibility of recall bias. In spite of choosing Sohag city and only two villages from Sohag district out of the 12 districts in the governorate, the results can be generalized to other areas in the Sohag governorate as their population has similar characteristics.

## Conclusions and recommendations

Unintended pregnancy is a public health problem in Sohag. Young women < 30, young husbands < 30, women working for cash, and low monthly income were risk factors for mistimed pregnancy, while women working for cash, living children ≥ 5, and the woman’s perception of her family size as higher than the ideal were risk factors for unwanted pregnancy. About two thirds of unintended pregnancies occurred in women who did not use contraceptives. Furthermore, unwanted pregnancy was associated with increased maternal complications during pregnancy, LBW, and decreased breastfeeding.

Some policy recommendations based on the findings of the present study are proposed that could be useful in developing a strategy to reduce unintended pregnancy among married women of the reproductive age in Sohag mainly: More logistic, educational, and health support should be directed to Upper Egypt; more health and educational care should be directed to rural areas; and improving the socio-economic level of women at risk of unintended pregnancy, especially the rural sector. In addition, raising community awareness of gender discrimination through wide use of mass media and educational sessions to change the cultural beliefs of preferring the male gender. More information is required about contraception and its complications and proper use, as well as better access to contraceptive services. Family planning programs should aim to raise awareness about the effective use and to reduce the unmet need for contraception.
